# Phosphoglycerate dehydrogenase induces glioma cells proliferation and invasion by stabilizing forkhead box M1

**DOI:** 10.1007/s11060-012-1018-x

**Published:** 2012-12-11

**Authors:** Jinlong Liu, Shaolei Guo, Qingzhi Li, Lixuan Yang, Zhibai Xia, Longjuan Zhang, Zhengsong Huang, Nu Zhang

**Affiliations:** 1 Department of Neurosurgery, The 1st Affiliated Hospital of Sun Yat-sen University, No 58, Zhongshan 2 Road, Guangzhou, Guangdong Province 510080 People’s Republic of China; 2 Laboratory Center of Surgery, The 1st Affiliated Hospital of Sun Yat-sen University, Guangzhou, Guangdong Province 510080 People’s Republic of China

**Keywords:** Phosphoglycerate dehydrogenase, Glioma, FOXM1, Protein stabilization

## Abstract

Phosphoglycerate dehydrogenase (PHGDH) is the first enzyme branching from glycolysis in the three-step serine biosynthetic pathway. Recent evidence has shown that PHGDH is amplified in human breast cancer and melanoma and plays a key role in cancer metabolism. However, PHGDH expression in glioma and a potential non-metabolic role in tumorigenesis have not been reported. We analyzed PHGDH levels in specimens from glioma patients and found that PHGDH, although negative in normal brain tissues, was highly expressed in astrocytic tumors and increasingly expressed in more aggressive cancer types. Inhibition of PHGDH expression in glioma cells downregulated the expression of VEGF, MMP-2, CHK2 and cyclin D1 and reduced glioma cell proliferation, invasion and tumorigenicity in vitro and in vivo. Interestingly, we found that the oncogenic transcription factor FOXM1 was also downregulated in PHDGH-silenced glioma cells. Using LC/LC MS analysis, we identified PHGDH as a novel binding partner of FOXM1. PHGDH interacted with and stabilized FOXM1 at the protein level, promoting the proliferation, invasion and tumorigenicity of glioma cells. Our data identified PHGDH as a potential prognostic marker of glial brain tumors and identified a non-metabolic role for PHGDH in glioma tumorigenesis, providing a novel angle of targeting the PHGDH–FOXM1 axis in future brain tumor therapy.

**Electronic supplementary material:**

The online version of this article (doi:10.1007/s11060-012-1018-x) contains supplementary material, which is available to authorized users.

## Introduction

Gliomas are the most common malignant tumor in the brain, and the overall prognosis for patients with malignant gliomas is poor. The cumulative 1-year survival rate of glioma patients is less than 30 % [1]. The most aggressive type of glioma, glioblastoma, accounts for approximately 50–60 % of all astrocytic tumors and 80 % of all malignant gliomas and has a median survival of only 15 months [1, 2]. Despite significant advances in neurosurgical techniques and the introduction of novel chemotherapies and aggressive multimodal treatments, the overall prognosis of glioma patients remains dismal [3, 4]. Translational and biological research has revealed that poorly circumscribed margins, invasion ability, and the uncontrolled proliferation of gliomas are contributing factors for the aggressiveness and high rates of recurrence [5, 6]. Therefore, understanding the molecular mechanisms of glioma invasiveness and proliferation is an urgent challenge in the development of new therapeutic strategies for this deadly disease.

The metabolic requirements of cancer cells differ from their normal counterparts, and altered metabolism is considered an emerging hallmark of cancer [7, 8]. Among a number of metabolic enzymes, one key enzyme for serine biosynthesis, PHGDH, has been reported in recent cancer biology studies [9]. Evidence has shown that PHGDH is amplified or overexpressed in a subset of melanoma and breast cancers [10, 11]. Depletion of PHGDH strongly inhibited the proliferation of a panel of breast cancer cells with amplified or overexpressed PHGDH [10]. However, the expression patterns of PHGDH in glioma and its molecular functions (aside from being a metabolic enzyme) have not yet been reported.

In this study, we showed that PHGDH was robustly expressed in a large proportion of astrocytomas, and its expression levels increased with tumor grade. Mechanistic investigations revealed that deprivation of PHGDH in glioma cells impaired proliferation and invasiveness. In nude mice injected with stable, PHGDH-silenced glioma cells, the overall survival was dramatically prolonged compared with the mice injected with wild-type cells. Furthermore, we identified PHGDH as a novel binding partner of FOXM1. This interaction stabilized FOXM1 protein levels and sequentially induced the expression of a series of oncogenes, such as MMP-2, VEGF, Chk2 and cyclin D1, which suggests that in addition to metabolic functions, PHGDH may have other biological roles in glioma tumorigenesis.

## Materials and methods

### Patient information and tissue specimens

A total of 132 paraffin-embedded glioblastoma samples were histopathologically and clinically diagnosed at our department from 2000 to 2006. Five paired patient samples, including tumor and tumor-adjacent tissues, were collected during surgery. Prior patient consent and approval from the Institutional Research Ethics Committee were obtained for the use of these clinical materials for research purposes.

### Cell lines and reagents

The glioma cells U87, U251, A172 and U373 were kindly provided by Dr. Suyun Huang, MD Anderson Cancer Center, Houston, TX. Human embryonic kidney 293T cells were obtained from the American Type Culture Collection, and normal human astrocyte cells were purchased from Lonza. The glioma cell lines and 293T cells were cultured in DMEM supplemented with 10 % fetal bovine serum (Invitrogen). The normal human astrocyte cells were maintained in Astrocyte Basal Medium (Lonza) plus AGM SingleQuots. Antibodies against PHGDH (sc-100317), FOXM1 (sc-500), MMP-2 (sc-13594), VEGF (sc-152), chk2 (sc-5278), and cyclin D1 (sc-717) were purchased from Santa Cruz Biotechnology; the antibody against actin was obtained from Sigma. The shRNA sequences targeting PHGDH were as follows: sh-PHGDH-1, CCGGAGGTGATAACACAGGGAACATCTCGAGATGTTCCCTGTGTTATCACCTTTTTT; and sh-PHGDH-2, CCGGCTTAGCAAAGAGGAGCTGATACTCGAGTATCAGCTCCTCTTTGCTAAGTTTTT. The control shRNA sequence targeted GFP. The fluorescent secondary antibodies Alexa Fluor 488 and Alexa Fluor 596 were from Invitrogen.

### Cell transfection and viral infection

To introduce short hairpin RNAs into the glioma cells, we used the PRNATU6.2 Lenti vector to generate viruses. Viral infections were serially performed, and stable cell lines expressing PHGDH RNAi(s) were selected using 0.5 μg/mL puromycin.

### Flow cytometry

The cells were harvested and fixed in ice cold 75 % ethanol before staining with propidium iodide (Sigma, 0.45 mg/mL), RNase (Sigma, 0.45 mg/mL), and 0.045 % Tween-20 (Sigma). The resuspended cells were analyzed for DNA content using a fluorescence-activated cell sorter (FACS; Vantage), and the data were processed using FACS Cell Quest software (Becton–Dickinson).

### Colony formation assays

Glioma cells carrying PHGDH or control shRNA were dissociated to single cell suspension in 0.6 % top agar medium and plated in triplicate in 60-mm dishes (300 cells per dish) that had been pre-coated with 1.2 % agar medium. The emergent colonies were stained with crystal violet 14 days later and counted.

### Immunohistochemical analyses

Immunohistochemical analyses were performed by staining tissue sections from paraffin-embedded specimens with an anti-PHGDH antibody. Nonspecific immunoglobulin (IgG) was used as a negative control. We quantitatively scored tissue sections according to the percentage of positive cells and staining intensity as previously described [12].

### Immunofluorescence staining

The cells were fixed with 4 % paraformaldehyde, permeabilized with phosphate-buffered saline (PBS) containing 0.1 % Triton X-100, and blocked with 1 % bovine serum albumin. Immunostaining was performed using the appropriate primary antibodies and stained with 4′,6-diamidino-2-phenylindole, anti-rabbit IgG conjugated with Alexa Fluor 488 or anti-mouse IgG conjugated with Alexa Fluor 596. The images were acquired using a scanning confocal microscope (Olympus FluoView FV1000).

### Real-time RT-PCR

Total RNA from cultured cells and frozen glioma tissues was extracted using TRIzol reagent (Invitrogen). Real-time RT-PCR assays were performed as described previously [12]. Each sample was analyzed in triplicate for the target gene and internal control.

### Western blotting, immunoprecipitation and MS

A Western blot analysis of cell lysates was performed with the antibodies previously described. The membranes were stripped and re-probed with an anti-beta-actin antibody as a loading control. For immunoprecipitation, the cells were lysed with sample buffer containing 62.5 mmol/L Tris–HCl (pH 6.8) and 10 % glycerol. Equal amounts of protein were mixed with antibodies and then incubated while being rotated overnight, followed by an agarose A/G bead (Millipore) addition. For MS analysis, the immunoprecipitates were separated on an SDS-PAGE gel, and various protein bands were collected, tryptically digested, and subjected to LC/LC MS analysis.

### Cell invasion assays

Cell invasion and migration assays were performed using a transwell system that incorporated a polycarbonate filter membrane (Corning, NY). To assess invasion, the filters were pre-coated with 10 μg of Matrigel (BD Biosciences, Franklin Lakes, NJ). A pretreated cell suspension (10^5^) in serum-free culture media was added to the inserts, and each insert was placed in the lower chamber filled with culture media containing 10 % FBS. After a 24-h incubation, the filters were fixed and stained with a 0.1 % crystal violet solution for 10 min. Five fields of adherent cells in each well were randomly imaged and counted.

### Intracranial tumor assay

Male, athymic, BALB/c nude mice were purchased from the Animal Center of Sun Yat-sen University. Glioma cells were intracranially injected into the nude mice as previously described. The animals were euthanized when they were moribund, and the remaining animals were euthanized 60 days after glioma cell injection. Each mouse brain was harvested, and tumor formation was determined by histological analysis. To analysis the tumor size and invasion, each of the tumor size was determined by three of the brain sections in which the tumor has the largest diameter. The invasion was counted when the invaded lesion is larger than 0.32 mm in 1 dimension of the slides. (The brain diameter of mouse/human is 12 mm/150 mm, and the clinical detectable invaded tumor on MRI is 5 mm. For mouse, the diameter should be 0.32 mm/12 mm = 0.03 compare with human 5 mm/150 mm = 0.03). The quantitative data of the in vivo tumor formation and invasion assay was shown in Fig. 5 and Supplementary Tables 3 and 4.

### Statistical analyses

Statistical analyses were performed using the SPSS software program (SPSS Standard version 13.0, SPSS Inc, Chicago, IL). The association between PHGDH expression and clinicopathologic grade was analyzed using Spearman rank test. The statistical significance of the correlation between PHGDH expression and disease-specific survival was estimated by the log-rank test. Evaluation of significant differences for in vitro data was determined using Student’s *t* test (two-tailed), one-factor of variance (ANOVA) analysis, two-factor of variance (ANOVA) analysis or Wilcoxon signed-rank test; and for in vivo data using the Mann–Whitney U test. Results are expressed as mean ± SD or ± SE from 3 independent experiments. A significance level set at *p* < 0.05.

## Results

### PHGDH was overexpressed in glioma samples at both the mRNA and protein levels

**Fig. 1:**
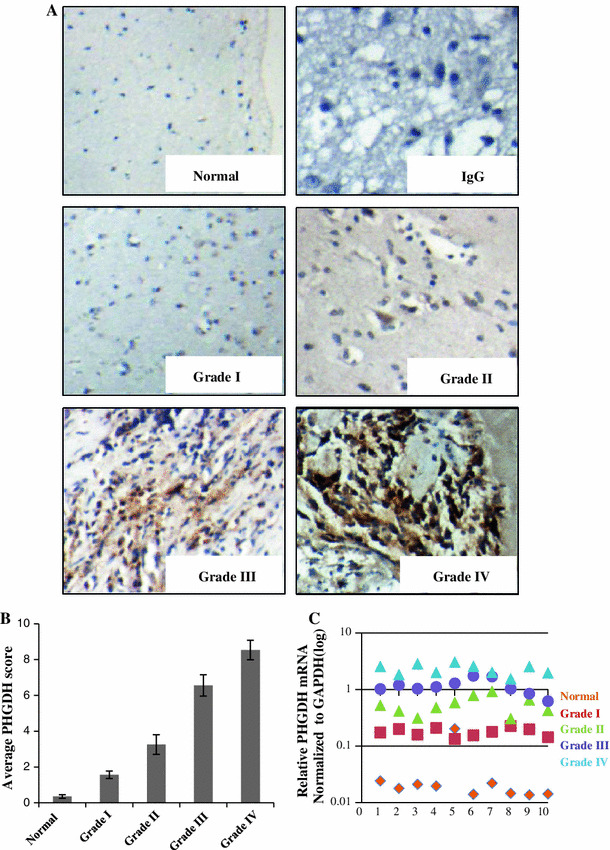
PHGDH was overexpressed in glioma samples at both the mRNA and protein levels. **a** Representing photes of parafin-embedded specimens of 132 primary astrocytoma specimens including WHO grade I–IV and 10 normal brain tissues stained by immunohidtochemistry using anti-PHGDH antibody. IgG were used as negative control. Amplifications are 200×. **b** Scoring of IHC staining samples. **c** Quantification PCR were used to detect the PHGDH mRNA expression of 40 glioma tissues (10 grade I astrocytomas, 10 grade II astrocytomas, 10 anaplastic astrocytomas and 10 glioblastomas) and 10 normal brain tissues. The expression level was normalized to GAPDH (log)

A total of 132 paraffin-embedded glioma specimens were obtained from patients who had undergone operations and were pathologically diagnosed in our department from 2001 to 2006 (the patient data are summarized in Supplementary Table 1); 10 normal brain tissue samples were also obtained. We detected PHGDH expression in these samples using a PHGDH-specific antibody. The positivity of PHGDH staining was increased in the more aggressive tumors, whereas the normal brain samples were PHGDH negative (Fig. 1a). We defined a Scoring Index (SI) for IHC staining samples: 0 (no positive tumor cells), 1 (<10 % positive tumor cells), 2 (10–50 % positive tumor cells), and 3 (>50 % positive tumor cells). The intensity of staining was graded according to the following criteria: 0 (no staining); 1 (weak staining = light yellow), 2 (moderate staining = yellow brown), and 3 (strong staining = brown). The staining index (SI) was calculated as staining intensity score x proportion of positive tumor cells (0, 1, 2, 3, 4, 6, 9). Cutoff values to define the high- and low-expression of PHGDH were chosen on the basis of a measurement of heterogeneity with the log-rank test statistic with respect to overall survival. Because univariate analysis demonstrated that the Cutoff value of 3 led to the highest significant difference with respect to overall survival in glioma between the respectively defined subgroup, an SI score >3 was taken to define tumors as high expression, and SI < 3 to define tumors as low expression of PHGDH. In the 132 tumor samples, 90 exhibited high PHGDH expression levels (68.2 %), and 42 exhibited low expression levels (31.8 %). In the statistical analyses, we determined that PHGDH expression levels highly correlated with the clinicopathological grade of the glioma samples (Fig. 1b; Supplementary Table 1; *p* < 0.0001). Next, we used Q-PCR to detect PHGDH mRNA expression levels in detect the PHGDH mRNA expression of 40 glioma tissues (10 grade I astrocytomas, 10 grade II astrocytomas, 10 anaplastic astrocytomas and 10 glioblastomas) and 10 normal brain tissues. As shown in Fig. 1c, the higher grade tumors showed elevated PHGDH mRNA levels, and there was a greater than 100-fold difference in the expression levels in GBMs compared with the normal brain. These data indicated that PHGDH was overexpressed in glioma, and its expression correlated with the glioma WHO grades.

### PHGDH as a prognostic marker for glioma

**Fig. 2:**
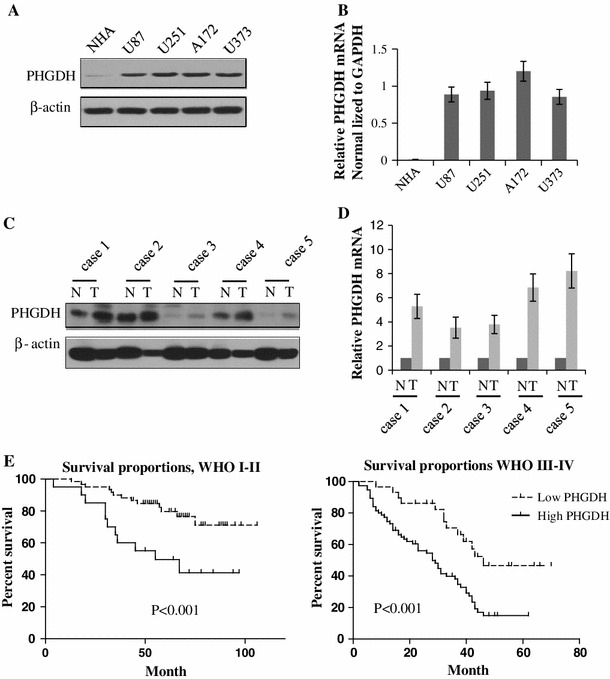
PHGDH as a prognostic marker for glioma. **a** Expression analysis of PHGDH protein in normal human astrocytes and astrocytoma cell lines by Western blotting. **b** Expression analysis of PHGDH mRNA in normal human astrocytes and astrocytoma cell lines by Q-PCR. **c** Differential expression of PHGDH protein in 5 primary astrocytoma specimens and their paired tumor adjacent tissues were detected by Western blotting. **d** Differential expression of PHGDH mRNA in the same 5 paired specimens in Fig. 2c was detected by Real-Time RT-PCR. Expression was normalized to GAPDH and SD was from 3 independent experiments.** e** Kaplan–Meier curves with univariate analyses (log rank) for low PHGDH expression versus high PHGDH expression in WHO I-II glioma patients and WHO III-IV glioma patients

To exclude the possibility that high PHGDH expression levels were due to cell types other than the astrocytomas, we analyzed PHGDH mRNA and protein expression levels in glioma cell lines and compared them with normal human astrocytes (NHA). As shown in Fig. 2a, b, PHGDH mRNA and protein was barely detectable in the NHA, whereas PHGDH mRNA and protein expression levels increased to different levels in the glioma cell lines. Because of the complexities of the genetic variation of these cell lines, we tested 5 paired clinical samples to confirm our findings. Not surprisingly, PHGDH mRNA and protein expression levels were elevated in all 5 paired samples (Fig. 2c, d). Kaplan–Meier curves indicated that in WHO grade I and II glioma patients, the 5-year survival was 82.3 % in the low-PHGDH expression group compared with 64.5 % in the high-PHGDH expression group (*p* < 0.001). In the WHO grade III and IV groups, the 5-year survival was 43.3 % in the low-PHGDH expression group compared with 18.5 % in the high-PHGDH expression group (*p* < 0.001). These data suggest that PHGDH is a prognostic marker for patients with glioma (Supplementary Table 2).

### Silencing of PHGDH attenuated glioma cell proliferation and invasion

**Fig. 3:**
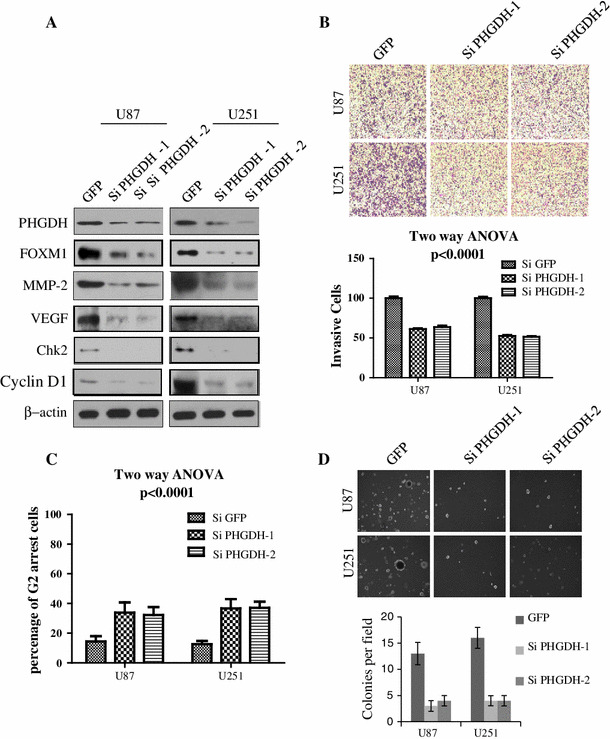
Silencing of PHGDH attenuated glioma cell proliferation and invasion. **a** Expression of series proliferation and invasion markers after PHGDH stable silencing in U87 and U251 cells was analyzed by western blot. **b** Upper, Photographs of transwell invasion assay of U87 and U251 cells that express shRNAs for control or PHGDH. Lower, the transwell invasion assay efficiency of the cells in** a**. Values are mean ± SD for triplicate samples and a two-way ANOVA was used to test the significance (*p* < 0.0001).** c** U87 and U251 cells that express shRNAs for control or PHGDH were subjected to flow cytometry assay. Values are mean ± SD for triplicate samples and a two-way ANOVA was used to test the significance (*p* < 0.0001).** d** Upper, photographs of soft agar colony formation of U87 and U251 cells that express shRNAs for control or PHGDH. Magnifications are 200×. Lower, the secondary neurosphereformation efficiency (spheres/cells plated) of the cells in A. Values are mean ± SD for triplicate samples and a Wilconxon test was used to test the significance (*p* < 0.05)

Recent evidence has shown that serine metabolism plays a pivotal role in human malignancy [13, 14]. As a key enzyme in serine de novo synthesis, inhibition of PHGDH inhibits tumor proliferation and reverses the malignant phenotype in breast cancer and menaloma [10, 11, 15]. To test whether this effect also exists in glioma cells, we established U87 and U251 cells with PHGDH stably silenced by 2 independent shRNAs referred to as PHGDH-1 and PHGDH-2. As shown in Fig. 3a, compared with the negative control, PHGDH-1 and PHGDH-2 significantly downregulated PHGDH expression levels in both cell lines. We next detected several oncogenes that were closely related to tumor proliferation and invasion in PHGDH-silenced U87 and U251 cells. The PHGDH shRNA dramatically reduced the expression levels of MMP-2 and VEGF, which are oncogenes important for tumor invasion and angiogenesis. Chk2 and cyclin D1, hallmarks of tumor proliferation, were also downregulated after PHGDH knockdown (Fig. 3a). Next, we examined whether PHGDH inhibition could impact glioma cell invasion using transwell chambers. As shown in Fig. 3b, PHGDH knockdown produced a significant reduction in the number of invasive cells. Compared with the control group, the number of invasive cells was reduced to 61.174 ± 1.25 % (PHGDH-1) and 63.86 ± 1.87 % (PHGDH-2) in U251 cells and 52.73 ± 0.96 % (PHGDH-1) and 51.43 ± 1.05 % (PHGDH-2) in U87 cells. To test the PHGDH silencing effects on proliferation, we analyzed the cell cycle distribution and tumorigenicity using flow cytometry and colony formation assays, respectively. After PHGDH inhibition, the U87 and U251 glioma cells exhibited a massive G2 arrest compared with the control cells. The G2-arrested cells increased to 33.9 ± 6.78 % (PHGDH-1) and 36.7 ± 6.22 % (PHGDH-2) in U87 cells and 32.20 ± 5.34 % (PHGDH-1) and 37.10 ± 4.10 % (PHGDH-2) in U251 cells, compare with 14.40 ± 3.57 % and 12.60 % ± 2.13 % in control U87 and U251 cells (Fig. 3c). PHGDH-silenced U87 and U251 cells both showed impaired tumorigenicity, as indicated by the observation that their colony formation ability was significantly decreased compared with the control groups (Fig. 3d). These results suggested that PHGDH contributed to glioma cell invasion and proliferation.

### PHGDH interacted with and stabilized FOXM1 in glioma cells

**Fig. 4:**
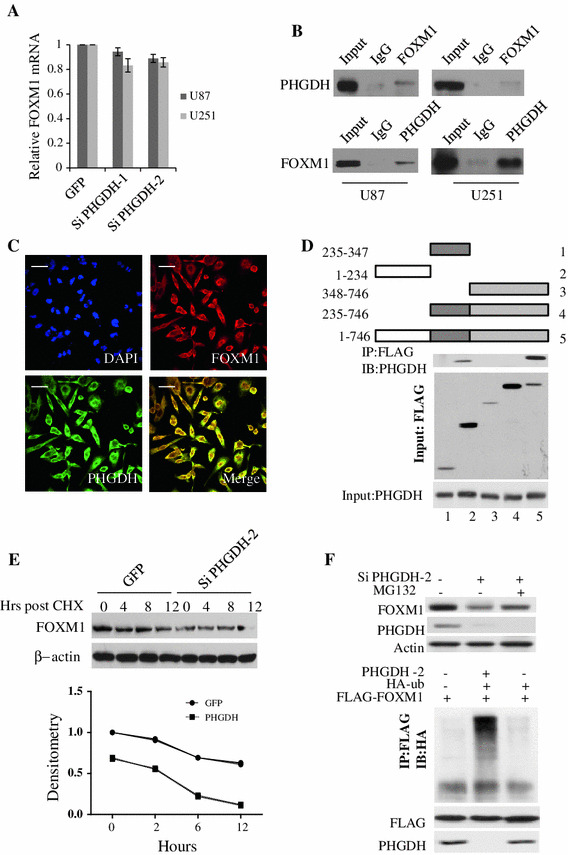
PHGDH interacts with and stabilize FOXM1 in glioma cells. **a** Expression of FOXM1 mRNA in U87 and U251 cells that express shRNAs for control or PHGDH was detected by Real-Time RT-PCR. Expression was normalized to GAPDH and SD was from 3 independent experiments (*p* = 0.1325). **b** Endogenous binding of PHGDH and FOXM1 were detected by using anti-PHGDH or anti-FOXM1 antibodies, respectively, in U87 and U251 cells. **c** U251 cells were plated in chambers precoated with poly-l-ornithine and fibronectin before stained with anti-FOXM1 and anti-PHGDH antibodies. Images were acquired using a scanning confocal microscope (Olympus FluoView FV1000). Scale bar for 20uM. **d** Flag-tagged FOXM1 fragments as indicated were transfected into U87 cells. 48 h post transfection, the co-IP were performed by using anti-FLAG antibody. **e** U251 cells that express shRNA for control or PHGDH were treated with cyclohexmide (50 μM) for 24 h before subjected to western blot analysis. The densitometry was acquired by Beckman Appraise, Beckman Instruments (*p* < 0.0001 in two way ANOVA).** f** Upper, control siRNA and PHGDH-2 siRNA were transfected into U87 glioma cells and 20 μM of MG132 were added as indicated 24 h post transfection. Total protein was analyzed 48 h post transfection by western blot. Lower, Flag-tagged FOXM1, HA-tagged Ubqutin and PHGDH-2 siRNA were co-transfected into U87 glioma cells as indicated. 48 h post transfection, co-IP experiments were done by using anti-Flag antibody

Interestingly, FOXM1 expression levels were also found to be decreased when PHGDH was silenced (Fig. 3a). FOXM1 is a well-known oncogene that induces cell proliferation, invasion and tumorigenecity [16–18]. In addition, key regulators of the cell cycle, cyclin D1 and chk2, are both direct transcriptional targets of FOXM1 [19, 20] We found that the mRNA levels of FOXM1 did not change when PHGDH was silenced (Fig. 4a). Using LC/LC MS, we identified PHGDH as a novel binding partner of FOXM1 (Supplementary Fig. 1). This result was confirmed through endogenous co-immunoprecipitation in U87 and U251 cells (Fig. 4b). To further validate this result, we performed immunofluorescence staining in U251 cell. As shown in Fig. 4c, both FOXM1 and PHGDH were strongly expressed in U251 cells, and these two proteins showed significant co-localization in the cytoplasmic but not nuclear region. Deletion fragments co-immunoprecipitation studies indicated that PHGDH interacts with the N-terminal region of FOXM1 (Fig. 4d). As N-terminal region of FOXM1 is known to induce FOXM1 degradation [21], we hypothesized that PHGDH could stabilize FOXM1 and protect it from degradation. Thus, we compared the degradation pattern of FOXM1 in PHGDH-knocked down U251 cells and control cells with cycloheximide treatment. In control cells, the FOXM1 density remained at 62.23 ± 11.64 % compared with 9.65 and 3.0 % in the PHGDH-silenced group after 12 h. In addition, the FOXM1 density curves showed faster degradation in PHGDH-silenced cells (Fig. 4e). The protesome inhibitor MG132 could reverse the effects of PHGDH silencing induced FOXM1 down regulation; and when PHGDH silenced by specific siRNA the ubiqutin conjugated FOXM1 increased dramatically compare with control group. From these data, we concluded that PHGDH could stabilize FOXM1 protein from degradation in glioma cells.

### PHGDH silencing inhibited glioma tumorigenicity in nude mice

**Fig. 5:**
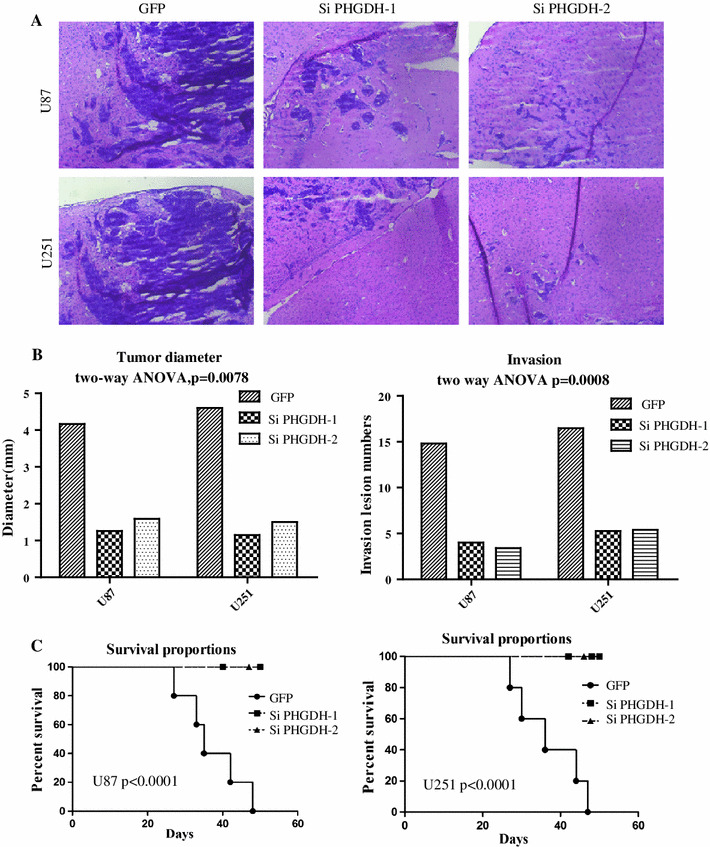
PHGDH silencing inhibited glioma tumorigenicity in nude mice. **a** 1*10^6^ U87 and U251 glioma cells that express shRNA for control or PHGDH were injected intracranially into nude mice as described previously. Brains were harvested when the animal were moribund; the remaining animals were killed 60 days after glioma-cell injection. Each mouse’s brain was fixed in 4 % formaldehyde, and embedded in paraffin. Tumor formation was determined by histologic analysis of H&E-stained sections. Magnification, 100×. **b** Statistical analysis of tumor size and invasion in intracranial tumor assay, a two-way ANOVA analysis of tumor diameter or invasion lesion numbers with respect to cell line (U87 versus U251) and shRNA expression (GFP, si-PHGDH-1, si-PHGDH-2, *p* < 0.0001). **c** Kaplan–Meier curves with univariate analyses (log rank) of nude mice Intracranial Tumor Assay, *p* < 0.0001

Finally, we tested the tumorigenicity after PHGDH silencing in nude mice. As previously described [22], we injected the control and PHGDH-silenced U87 and U251 cells into the brains of nude mice. In the control group, both cell lines resulted in the death of all 5 mice within 50 days, while the si-PHGDH-1 and si-PHGDH-2 groups showed a prolonged survival time (Log-rank tests of overall survival *p* < 0.0001). The mouse brain section staining confirmed that both the tumor size and invasion were dramatically inhibited after PHGDH silencing (Supplementary Tables 3 and 4) and a two-way ANOVA analysis of tumor diameter or invasion lesion numbers with respect to cell line (U87 versus U251) and shRNA expression was shown the results were significant (Fig. 5a, b, c).

## Discussion

Cancer cells are known to import and consume increased amounts of glucose compared with normal cells [23]. In brain tumors, the best evidence that metabolic enzymes play a causative role comes from the discovery of the IDH1 and IDH2 mutations [24]. More than 70 % of Stage II–III astrocytoma or oligodendroglioma tumors have mutations in either IDH1 or IDH2 [25]. PHGDH, the gene encoding the enzyme that controls the flux from glycolysis into the serine biosynthesis pathway, is a candidate oncogene. In breast cancer, elevated PHGDH levels are more frequently found in estrogen receptor-negative and basal-like tumors. RNAi-mediated depletion of PHGDH has little effect on the viability of untransformed breast epithelial cells or breast cancer cells without PHGDH amplification/overexpression [10]. Melanoma, however, with 39 % of samples exhibiting some form of copy number gain, had the highest frequency of PHGDH amplification among tumor types that were analyzed [11]. Consistent with the results in breast cancer, knockdown of PHGDH inhibited the growth of melanoma cell lines that harbored the PHGDH amplification but had no effect on lines lacking the amplification [11]. Based on these results, PHGDH may be an ideal drug target for cancer therapy. However, the expression of PHGDH in glioma and its roles in addition to the Warburg effect has not yet been demonstrated.

Genome-wide gene expression profiling has independently and consistently identified FOXM1 as one of the most commonly upregulated genes in human solid tumors [26, 27]. Depletion of FOXM1 in glioma cells resulted in a reduction in cell proliferation and anchorage-independent colony formation on soft agar. Furthermore, FOXM1 knockdown was associated with a decreased expression of the cell cycle proteins cyclin A2, cyclin B1, and Cdc25 phosphatases and increased expression levels of the cell cycle inhibitors p21Waf1/Cip1 and p27Kip1 [28]. Other evidence has shown that suppression of FOXM1 leads to a reduction in MMP-2 and MMP-9 expression levels in pancreatic cancer cells, which is associated with an overall decrease in cancer cell migration, invasion and angiogenesis [29]. Reports have shown that FOXM1 is transcriptionally regulated by Gli1 in basal cell carcinomas [30]; however, whether FOXM1 is regulated at the protein level has not been reported.

To explore PHGDH expression in gliomas, we performed immunohistochemical analysis on 132 paraffin-embedded glioma specimens ranging from WHO grade I to grade IV. We found that PHGDH expression levels correlated with the tumor grade, whereas normal brains showed no detectable PHGDH staining. The results were consistent with the PHGDH expression pattern in melanoma and breast cancer. Moreover, we identified PHGDH as a potential prognostic marker for glioma patient cumulative survival. Further studies showed that PHGDH interacts with N-terminal of FOXM1, stabilized FOXM1 from ubiqutintion induced protesome degradation in glioma cells, suggesting that PHGDH may be a regulator of FOXM1, and that PHGDH has additional biological functions in addition to those as a metabolic enzyme. Further work will be focus on how PHGDH affects the FOXM1 N-terminal induced degradation process. Recent reports have shown that another metabolic enzyme, embryonic pyruvate kinase M2, can induce beta-catenin translocation upon EGFR activation, which is another example of a metabolic enzyme playing a non-metabolic role in tumorigencity [31]. We inferred that PHGDH is a direct upstream regulator of FOXM1, and targeting PHGDH not only inhibited the aberrant metabolism of tumor cells but also depleted FOXM1 expression, thus attenuating tumor proliferation and invasion.

In conclusion, we report that PHGDH is a novel prognostic marker in glioma patients. Inhibition of PHGDH in glioma cells significantly decreased cell proliferation, invasion and tumorigenicity. Mechanistic studies revealed that PHGDH could interact with FOXM1 and stabilize it at the protein level. The PHGDH-FOXM1 axis may be an ideal drug target for future brain tumor treatments.

## Electronic supplementary material

Below is the link to the electronic supplementary material.

Supplementary material 1 (DOCX 602 kb)
