# Correction to: Phosphoglycerate dehydrogenase induces glioma cells proliferation and invasion by stabilizing forkhead box M1

**DOI:** 10.1007/s11060-025-04960-y

**Published:** 2025-02-14

**Authors:** Jinlong Liu, Shaolei Guo, Qingzhi Li, Lixuan Yang, Zhibai Xia, Longjuan Zhang, Zhengsong Huang, Nu Zhang

**Affiliations:** 1 Department of Neurosurgery, The 1st Affiliated Hospital of Sun Yat-sen University, No 58, Zhongshan 2 Road, Guangzhou, 510080 Guangdong Province People’s Republic of China; 2 Laboratory Center of Surgery, The 1st Affiliated Hospital of Sun Yat-sen University, Guangzhou, 510080 Guangdong Province People’s Republic of China

**Correction to: J Neurooncol (2013) 111:245–255** 10.1007/s11060-012-1018-x

In this article the content of the bottom, left-hand image in Fig. 5a overlaps with a substantial part of the top, left-hand image. The correct Fig. 5a is shown below.

**Figure:**
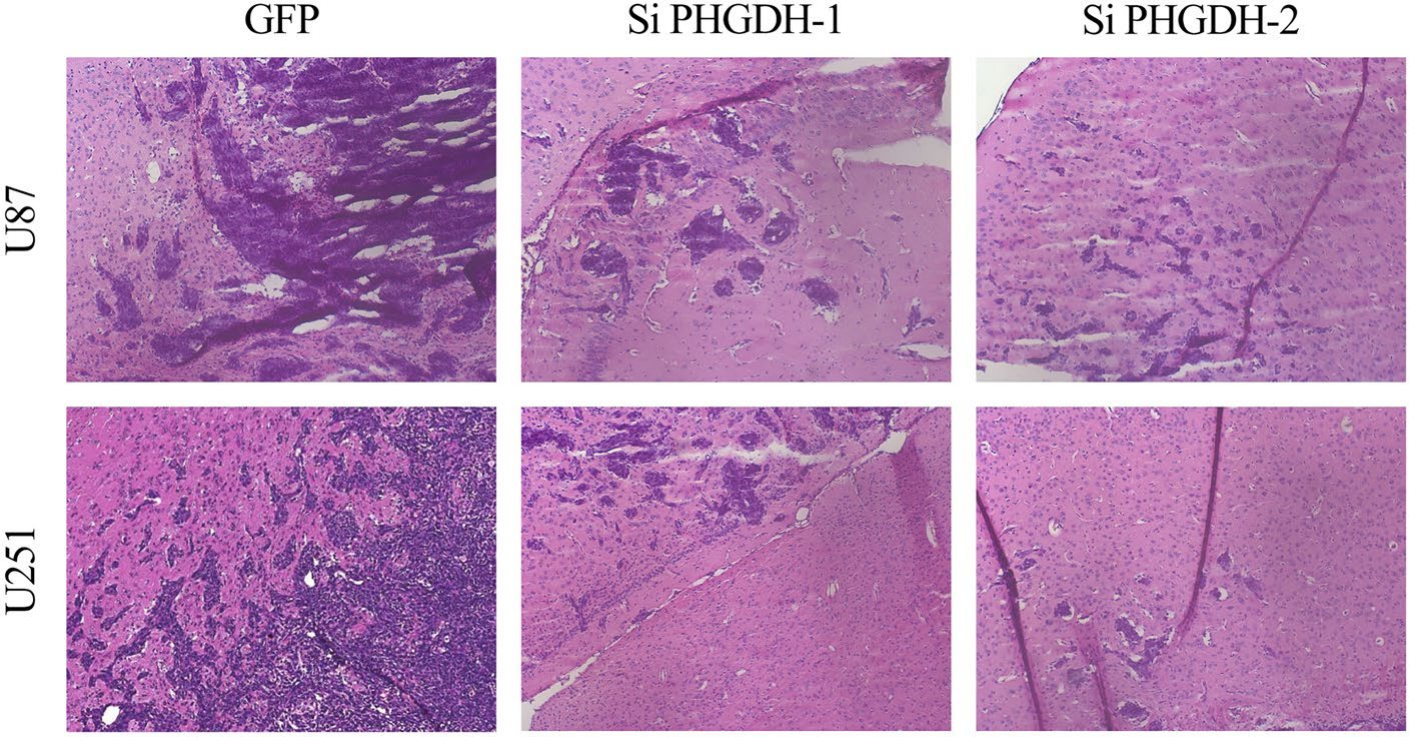
Correct Figure 5A

