# Supplementary material for: Phosphoglycerate dehydrogenase induces glioma cells proliferation and invasion by stabilizing forkhead box M1
Source: J Neurooncol. 2012 Dec 11;111(3):245–55. doi: 10.1007/s11060-012-1018-x (PMC3565087; doi:10.1007/s11060-012-1018-x)
Supplement: Supplementary file 1 — Supplementary material 1 (DOCX 602 kb) [file 11060_2012_1018_MOESM1_ESM.docx]

**Amplification of Phosphoglycerate Dehydrogenase Induces Glioma Cells Proliferation and Invasion by Stabilizing Forkhead Box M1**

**Journal of neuro-oncology**

Jinlong Liu^1,3^, Shaolei Guo^1,3^, Qingzhi Li^1^, Lixuan Yang^1^, Longjuan Zhang^2^, Zhengsong Huang^1^* and Nu Zhang^1^*

^1^Department of Neurosurgery, The 1^st^ Affiliated Hospital of Sun Yat-sen University, Guangzhou, Guangdong Province, 510080, PR China

^2^Laboratory Center of Surgery, The 1^st^ Affiliated Hospital of Sun Yat-sen University, Guangzhou, Guangdong Province, 510080, PR China

^3^These authors contributed equally to this work.

*Correspondence Authors

Nu Zhang, MD.

Department of Neurosurgery

The 1^st^ Affiliated Hospital of Sun Yat-Sen University

No 58, Zhongshan 2 Road

Guangzhou, Guangdong Province 510080

Fax: 86-20-8733-1655

Email: [zhangxiaonu@gmail.com](mailto:zhangxiaonu@gmail.com)

**Supplementary Table1.**

The relationship between PHGDH protein expression and patient clinicopathologic characteristics

| expression | Low  (n = 42) | High  (n = 90) | *P |
| --- | --- | --- | --- |
| Age (y) |  |  | 0.02 |
| <35 | 26 | 42 |  |
| ≥35 | 16 | 48 |  |
| Gender |  |  | 0.021 |
| Male | 20 | 62 |  |
| Female | 22 | 28 |  |
| Stage |  |  | 0.000 |
| Ⅰ | 10 | 5 |  |
| Ⅱ | 20 | 15 |  |
| Ⅲ | 8 | 42 |  |
| Ⅳ | 4 | 28 |  |

**Supplementary Table 2.**

Cox’s multivariate analysis

| Terms Risk ratio | Risk ratio | 95% Confidence  interval | P |
| --- | --- | --- | --- |
| Gender (male) | 1.516 | 0.948-2.501 | 0.416 |
| Age (>35 y) | 1.361 | 0.647-2.865 | 0.324 |
| Stage(Ⅲ, Ⅳ) | 5.431 | 2.981-9.896 | 0.000 |
| PHGDH(immunohistochemicallypositive) | 2.518 | 1.167-5.431 | 0.018 |

**Supplementary Table3. Tumor diameter (mm) of intracranial tumor formation assay**

|  | GFP  (Mean, 95% CI) | PHGDH-1  (Mean,95% CI) | PHGDH-2  (Mean, 95% CI) |
| --- | --- | --- | --- |
| U87 | 4.166±0.6378  (3.813-4.519) | 1.26±0.4240  (1.025-1.495) | 1.149±0.4190  (0.9771-1.322) |
| U251 | 4.602±0.7881  (4.166-5.038) | 1.591±0.3110  (1.359-1.823) | 1.501±0.4367  (1.259-1.743) |

**Supplementary Table4. Invasion numbers of intracranial tumor formation assay**

Invasion was defined as tumor lesion that located near but not continues with the primary injection site. The invasion was counted when the invaded lesion is larger than 0.32mm in 1 dimension of the slides. (The brain diameter of mouse/human is 12mm/150mm, and the clinical detectable invaded tumor on MRI is 5mm. For mouse, the diameter should be 0.32mm/12mm=0.03 compare with human 5mm/150mm=0.03).

|  | GFP  (Mean, 95% CI) | PHGDH-1  (Mean, 95% CI) | PHGDH-2  (Mean, 95% CI) |
| --- | --- | --- | --- |
| U87 | 14.80±4.144  (12.51-17.09) | 4.000±1.464  (3.189-4.811) | 3.405±1.676  (3.405-5.262) |
| U251 | 16.47±3.796  (14.36-18.57) | 5.267±1.534  (4.417-6.116) | 5.400±2.501  (4.015-6.785) |

**Supplementary Fig1**

**
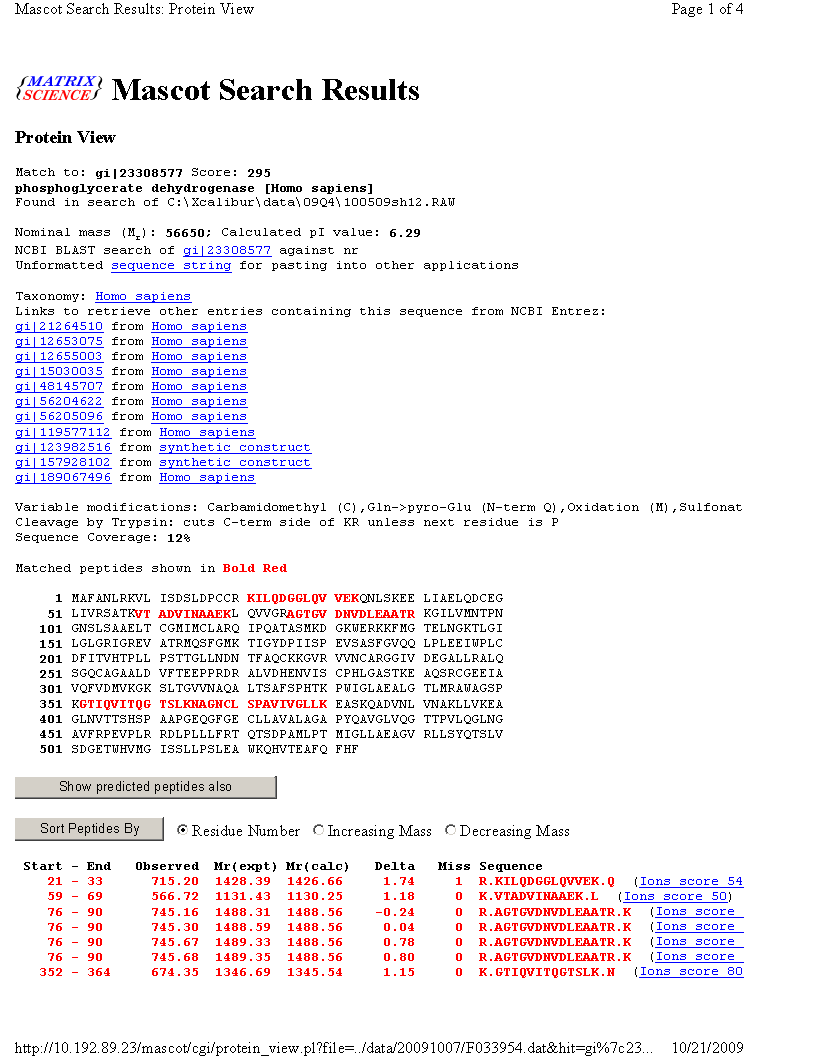
**

**
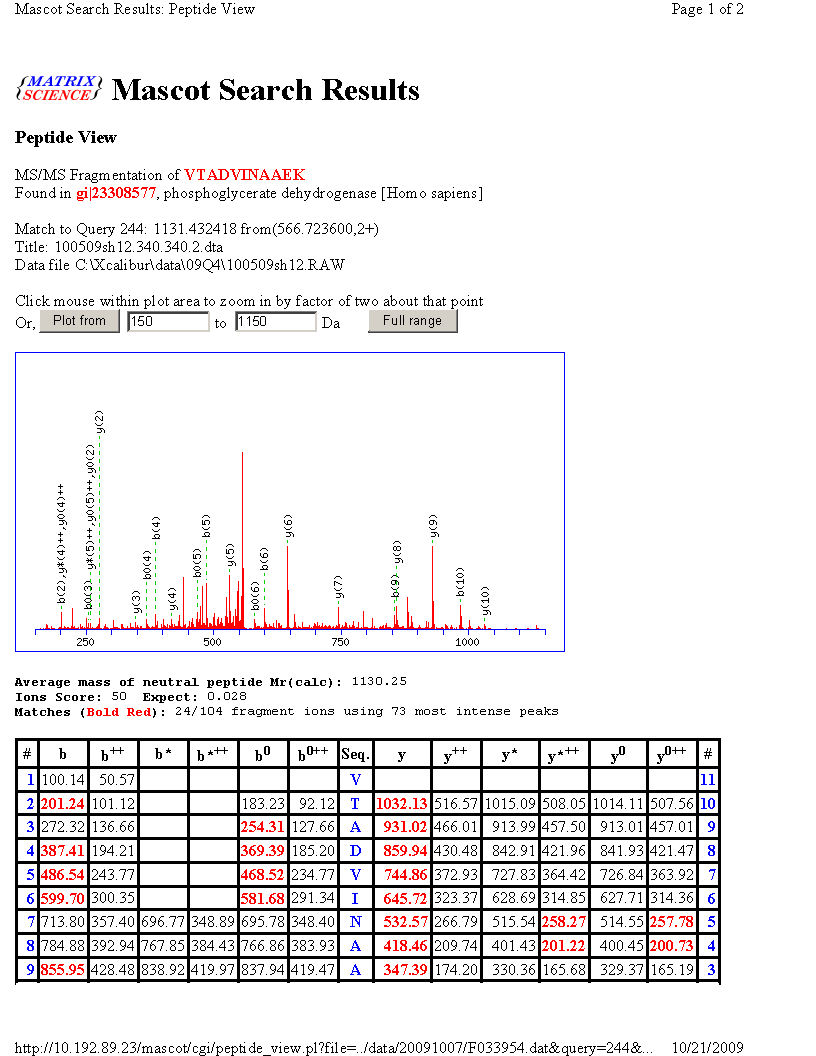
**

**
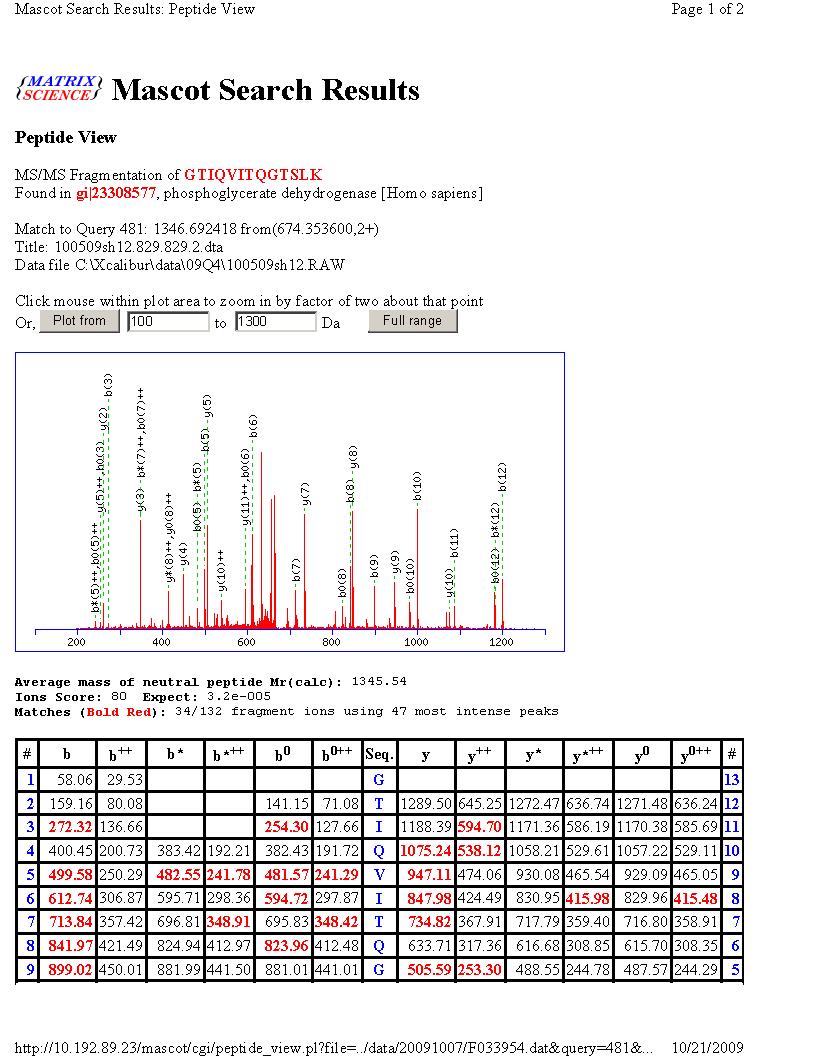
**

**
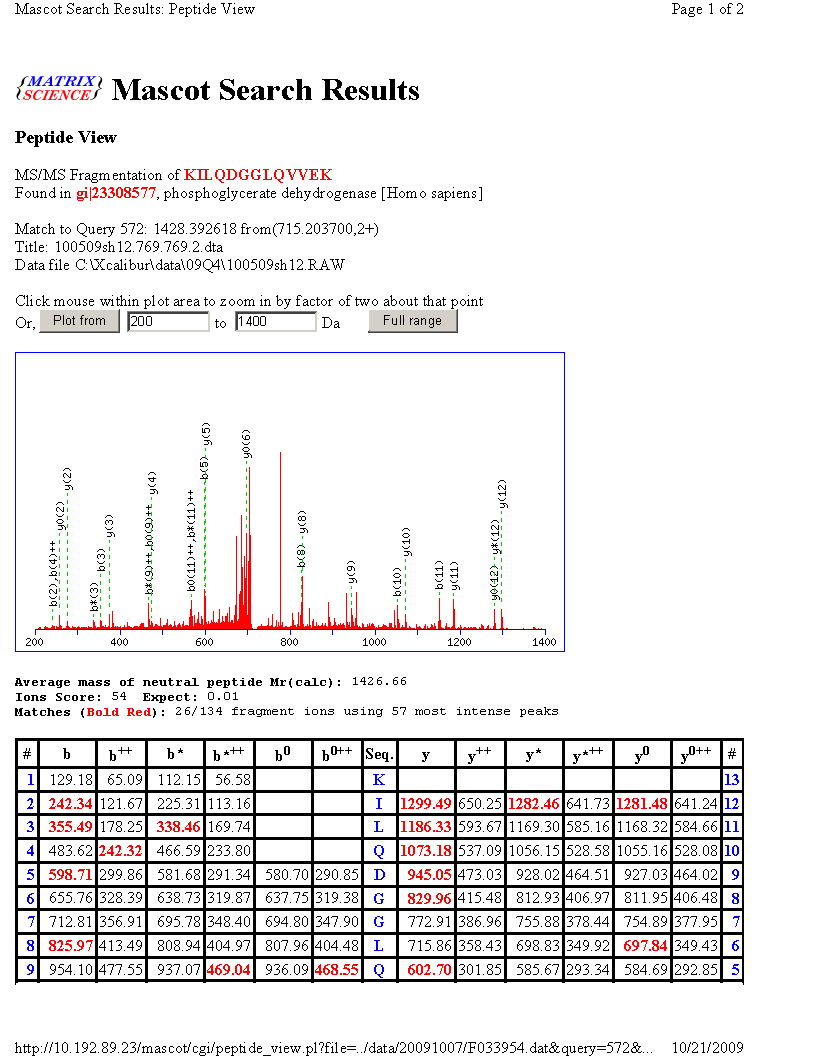
**

**
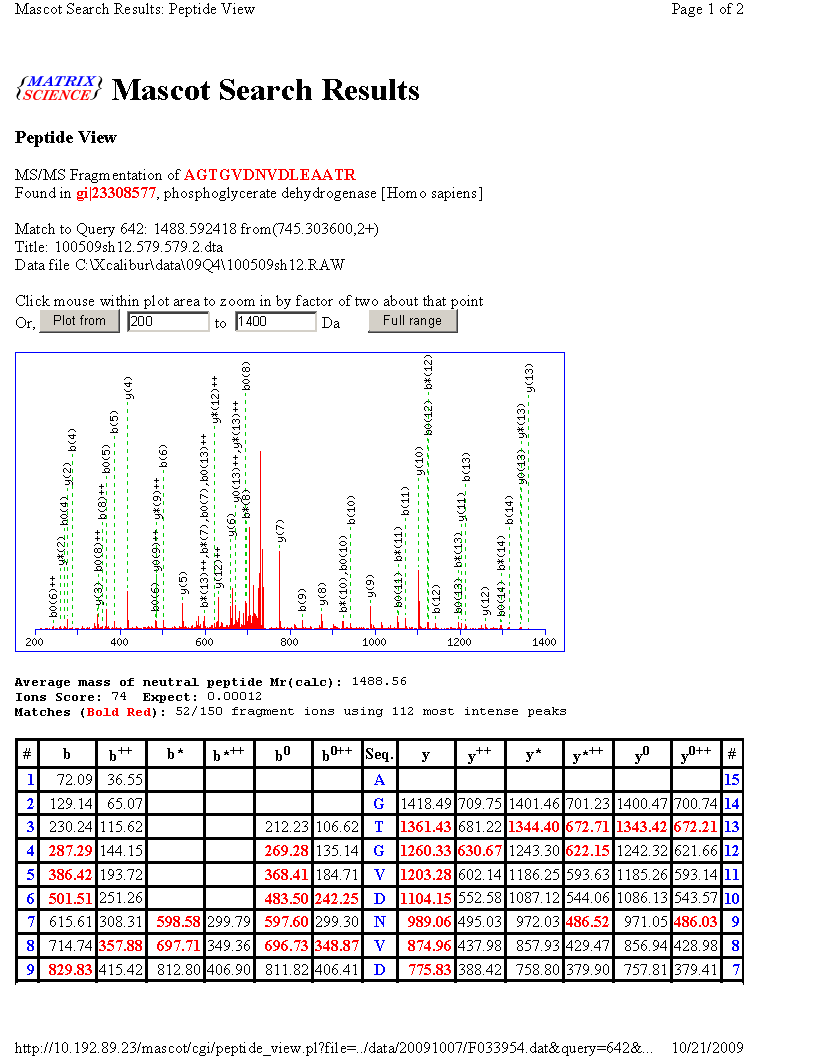
**

**
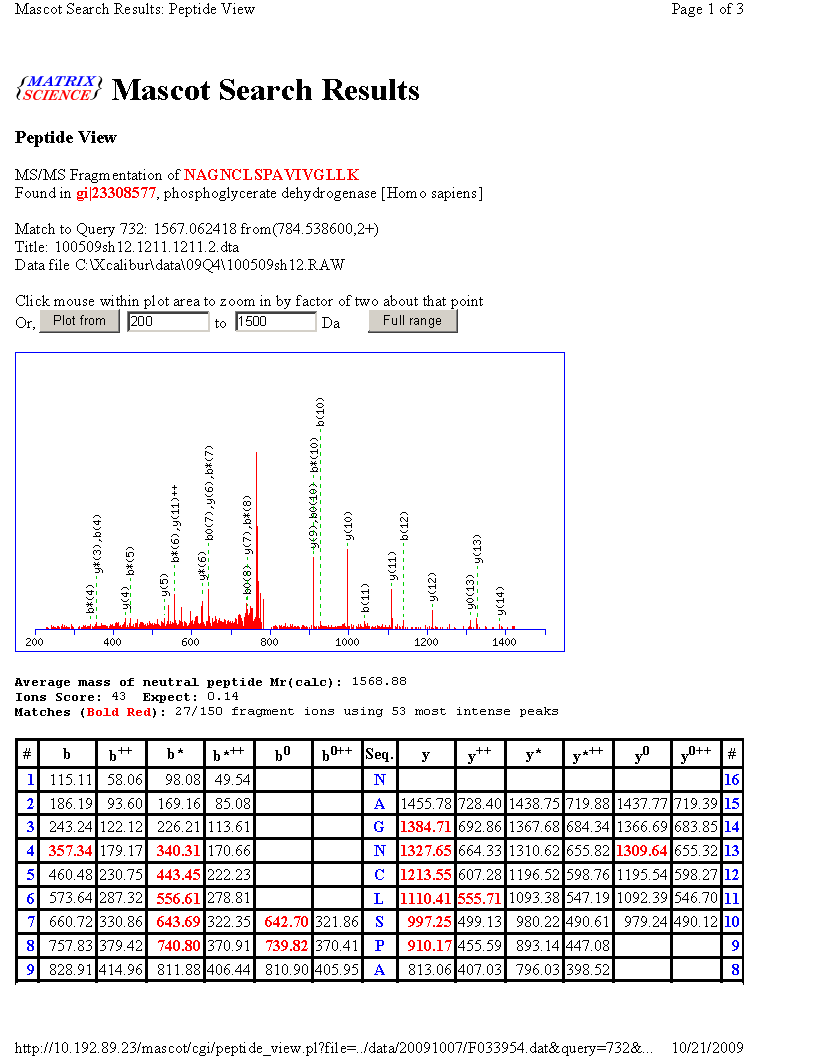
**
